# A Novel Numerical Model of Gelant Inaccessible Pore Volume for In Situ Gel Treatment

**DOI:** 10.3390/gels8060375

**Published:** 2022-06-13

**Authors:** Jianqiao Leng, Xindi Sun, Mingzhen Wei, Baojun Bai

**Affiliations:** 1 Department of Geosciences and Geological and Petroleum Engineering, College of Engineering and Computing, Missouri University of Science and Technology, Rolla, MO 65401, USA; jl3kf@umsystem.edu; 2 Department of Physics and Pre-Engineering, Slippery Rock University of Pennsylvania, Slippery Rock, PA 16057, USA; xindi.sun@sru.edu

**Keywords:** IAPV, gelant, placement, rheology, retention

## Abstract

Inaccessible pore volume (IAPV) can have an important impact on the placement of gelant during in situ gel treatment for conformance control. Previously, IAPV was considered to be a constant factor in simulators, yet it lacked dynamic characterization. This paper proposes a numerical simulation model of IAPV. The model was derived based on the theoretical hydrodynamic model of gelant molecules. The model considers both static features, such as gelant and formation properties, and dynamic features, such as gelant rheology and retention. To validate our model, we collected IAPV from 64 experiments and the results showed that our model fit moderately into these lab results, which proved the robustness of our model. The results of the sensitivity test showed that, considering rheology and retention, IAPV in the matrix dramatically increased when flow velocity and gelant concentration increased, but IAPV in the fracture maintained a low value. Finally, the results of the penetration degree showed that the high IAPV in the matrix greatly benefited gelant placement near the wellbore situation with a high flow velocity and gelant concentration. By considering dynamic features, this new numerical model can be applied in future integral reservoir simulators to better predict the gelant placement of in situ gel treatment for conformance control.

## 1. Introduction

Reservoir heterogeneity is one reason for low oil recovery and early excessive water production. To reduce excessive water production and to improve sweep efficiency, many technologies, including polymer flooding and foam flooding, have been widely applied over the past several decades [1]. One of the most popular treatment methods is to inject gels to reduce the flow capacity of channels or fractures and divert the following fluid (i.e., water) to un-swept oil zones [2,3,4,5,6].

**Figure 1:**
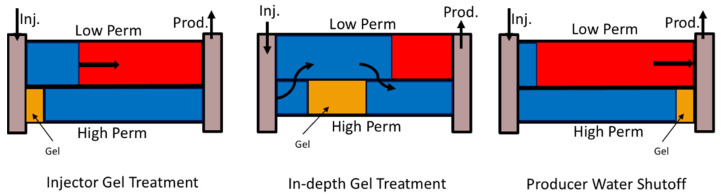
Application of gel treatment.

In situ gel is a widely applied gel treatment agent that can efficiently improve the injection profile and sweep efficiency during the driving phase [3,7,8]. The basic operational process for this technology is to pump the polymer solution and crosslinker as a mixture solution, called gelant, into the formation. Then, the well is shut in for a certain time to ensure in situ gelation sufficiently takes place. During this period, the slug of gel as a permeability modifier or barrier in the preferential water channel forms. Finally, the well opens and the subsequent water drive is diverted to the un-swept zone. Based on the location of the gel placement, the treatment can be categorized by profile control at injection wells, in-depth flow diversion, and water shutoff at production wells, as shown in Figure 1.

The results from the gel treatment, however, strongly depend on the placement of gelant [9]. The success experience favors the cases where gelant is preferentially or selectively placed in high permeability channels [10,11,12].

Many factors that benefit the gelant placement of gelant have been previously studied [13,14,15,16]. Inaccessible pore volume (IAPV) is one of the beneficial factors. With extensive experiments, [17,18] found that some pores in porous media are inaccessible to the polymers due to large size of polymer molecules. Since then, many studies have been applied to learn more about the factors that influence IAPV.

Szabo [19] found that the IAPV was much higher in the low K zone than in the medium or high K zones, and that the IAPV decreased when the post water flooding time increased. Zhang and Seright [20] also observed this phenomenon, stating that the desorption during a long period of post-water coreflooding reduced the IAPV. Shah et al. [21] and Gupta and Trushenski [22] found that the IAPV decreased when polymer concentrations increased. However, Ilyasov et al. [23] stated that their IAPV results were strongly influenced by the increased polymer retention due to increased polymer concentrations.

Liauh et al. [24] studied the effect of hydrodynamic exclusion on large molecules near the pore wall on IAPV. Thus, they proposed a relationship between pore/polymer size ratio and IAPV. Pancharoen et al. [25] studied IAPV experimentally and numerically, concluding that the mole weight of the polymer could positively influence IAPV and that salinity could negatively influence IAPV. Lotsch et al. [26] and Gilman and MacMillan [27] stated that measurement methodology, polymer type, and injection operations significantly influenced the results.

Sorbie [28] and Omari et al. [29] stated that the depletion layer in complex pore networks were also attributed to IAPV. Based on their theory, Chauveteau and Zaitoun [30] and Ferreira and Moreno [31] derived IAPV as a function of apparent viscosity, which indicated the dynamic variation of IAPV occurred during polymer flow in porous media. However, their model also assumed the low viscosity of polymer and could not quantify the IAPV under shear rate in shear thickening period.

Manichand and Seright [32] and Swadesi et al. [33] summarized the experimental results of IAPV and found that the IAPVs had a large variation and were very inconsistent because of interactive influential factors and lab operational properties. Fedorov et al. [34] also stated that the theoretical result of IAPV greatly differed from the lab results. Therefore, the IAPV model should contain the effects of influential factors and the dynamic properties during the polymer flow. However, no eligible numerical models have been reported and the reservoir simulators (i.e., CMG, ECLIPSE, VIP, and IORCoreSim) only considered a constant value for IAPV [35]. The drawbacks of the previous models are that once the IAPV is input into the simulator, the value does not change with the gel and rock properties, which may vary greatly during gelant placement. Therefore, the results of gelant placement could be erroneously simulated due to previous ineligible IAPV models.

This study sought to derive a numerical model based on the theoretical model, which is necessary for the consideration of both static and dynamic properties during gelant placement. Herein, this paper presents a validation with 64 sets of experimental results. We also discuss sensitivity studies and the impact of dynamic properties on IAPV, as well as the consequent impact on gelant placement. This new numerical model can be applied in future integral reservoir simulators to better predict the gelant placement of in situ gel treatment for conformance control with a comprehensive consideration of IAPV.

## 2. Methodology

### 2.1. Governing Equation of Inaccessible Pore Volume

A gelant molecule has a chain structure. When a chain is confined in a pore space, entropy decreases because movement is restricted. Thus, the gelant molecules tends to stay outside of pores in higher entropy regions. Because the repulsion of like charges on partially hydrolyzed polyacrylamide (HPAM) causes the gelant chain to deviate from the random state idealization, to simplify the problem we assumed that the gelant chain segments moved randomly and had a random configuration in the solution.

Based on the random state idealization, we determined the following calculations considering the equilibrium distribution of gelant in complex pore networks. The probability, P, of the *n*-th segment of a random distributed gelant molecule with segment length *L* at location (*x*) was derived by Dimarzio [36] using Equation (1).

(1) $$\frac{\partial P\left(x\right)}{\partial n}=\frac{L^{2}}{6}\nabla^{2}P\left(x\right)$$

The accessible pore volume (APV), which is 1-IAPV, equals the fraction of pore volume that contains gelant divided by the whole pore volume. It can be simplified as the fraction of probability of finding gelant in specific location considering restriction boundaries divided by that considering infinite boundaries. Thus, we can calculate APV by generalizing the Equation (2) as dimensionless. To do this, we divided Equation (1) by $P_{0}$, which is the probability of having a gelant chain in the specific pore when there is no boundary. Thus, Equation (2) equals the APV.

(2) $$\mathrm{APV}=\frac{\partial \left(\frac{P\left(x\right)}{P_{0}}\right)}{\partial n}=\frac{L^{2}}{6}\nabla^{2}\left(\frac{P\left(x\right)}{P_{0}}\right)$$

If the pore geometry is considered as infinite cylinders with radius *r*, we find boundary conditions in Equation (3).

(3) $$B.C.=\{\begin{array}{c} \frac{\partial \left(\frac{P\left(x\right)}{P_{0}}\right)}{\partial n}=0\;\;\;\;\;if\;\;\;n>0,\;\;\;x=0\\ \frac{P\left(x\right)}{P_{0}}=0\;\;\;\;\;\;\;\;if\;\;\;\;n>0,\;\;\;x=r\\ \frac{P\left(x\right)}{P_{0}}=1\;\;\;\;\;\;\;\;\;\;\;\;\;\;\;\;\;\;\;\;\;\;\;if\;\;\;\;n=0 \end{array}$$

Taking the average from *x* = 0 to *x* = *a*, we can solve the above equations. The solved distribution of gelant molecules or the APV is shown using Equation (4).

(4) $$\mathrm{APV}=1-\mathrm{IAPV}=4\overset{\infty }{\underset{m=1}{\sum }}\frac{1}{\beta_{m}^{2}}exp(-\beta_{m}^{2}\times {\left(\frac{R}{r}\delta_{1}\right)}^{2})$$

where *R* is the radius of gyration of the gelant molecule; r is pore radius; $\rho_{m}$ is the *m*-th root of the Bessel function of first kind of zeroth order; δ_1_ is tuning factor used for a different gelant and rock system. The IAPV is calculated using 1 − APV.

### 2.2. Derivation of Gelant Radius

Calculating the radius of gyration of the gelant molecule is not simple because the radius can significantly vary during gelant that flows in porous media. The conventional method [24] ignores the size variation of gelant molecules and assumes that the gelant chains are rigid.

A more reasonable method is to apply the hydrodynamic radius of gelant molecules in porous media. To simplify the problem, Lohne et al. [37] considered a swelling factor on a conventional rigid gelant model and gave an Equation (5) to calculate viscosity of a dilute suspension of rigid spheres based on the Stokes–Einstein equation:

(5) $$\frac{\mu }{\mu_{s}}=\left(1+S_{f}/2\times \frac{C_{p}}{\rho_{p}}\right)/{\left(1+S_{f}\times \frac{C_{p}}{\rho_{p}}\right)}^{2}$$

where μ is viscosity under shear; $\mu_{s}$ is static viscosity under zero shear rate (e.g., plateau viscosity); $S_{f}$ is swelling factor in dimensionless; $C_{p}$ is mass concentration; $\rho_{p}$ is gelant solution density.

With Einstein’s first order approximation and assumption of $C_{p}\to 0$, we have Equation (6).

(6) $$\rho_{p}=2.5\times S_{f}/\mu_{0}$$

where $\mu_{0}$ is intrinsic viscosity that can be expressed using Equation (7) based on Hiemenz and Lodge [38].

(7) $$\mu_{0}=\underset{C_{p}\to 0}{\lim}\left(\mu -\mu_{s}\right)/\left(C_{p}\mu_{s}\right)$$

Hirasaki and Pope [39] derived a gelant density equation based on dense spherical radius and gelant mole weight, as shown in Equation (8).

(8) $$\rho_{p}=\frac{M_{w}}{N_{A}}/\left(\frac{4\pi R_{h}^{3}}{3}\right)$$

where $M_{w}$ is mole weight; $N_{A}$ is Avogadros’ number; and $R_{h}$ is hydrodynamic radius.

Combining Equation (6) and Equation (8), we have Equation (9).

(9) $$R_{h}={\left(\frac{3}{10\pi N_{A}}\right)}^{\frac{1}{3}}\times {\left(\mu_{0}*M_{w}\right)}^{\frac{1}{3}}$$

To consider the effect of dynamic shear rate, we need to consider the apparent viscosity under shear instead of intrinsic viscosity. Therefore, we need to integral both side of Equation (7) and substitute it to Equation (9). Then, we have Equation (10), where $\delta_{2}$ is the tuning factor.

(10) $$R_{h}={\left(\frac{3}{10\pi N_{A}}\right)}^{\frac{1}{3}}*{\left(\frac{\mu -\mu_{s}}{\mu_{s}}*M_{w}\times \left(\ln\left(C_{p}\right)+\delta_{2}\right)\right)}^{\frac{1}{3}}$$

The apparent viscosity is calculated using Equation (11), which is a simplified dual power law model from Delshad et al. [40] and Zechner et al. [41].

(11) $$\mu =\{\begin{array}{c} \mu_{s}{\left(\frac{\gamma }{\gamma_{min}\;}\right)}^{n_{thin}-1}+\mu_{max}{\left(\frac{\gamma }{\gamma_{max}\;}\right)}^{n_{thick}-1},\;\gamma_{low}\leq \gamma \leq \gamma_{high}\\ \mu_{s}\;,\;\gamma <\gamma_{low}\\ \mu_{max}\;,\;\gamma >\gamma_{high} \end{array}\}$$

where γ is effective shear rate; $\gamma_{min}$ is the onset of shear thinning period; $\gamma_{max}$ is the maximum shear rate that gelant can take without degradation; $\mu_{max}$ is the viscosity measured at this maximum shear rate; and $n_{thin}$ and $n_{thick}$ are the shear thinning and shear thickening coefficients, respectively.

In the simulation, the effective shear rate γ is calculated using Equation (12) [42].

(12) $$\gamma_{e}=C\;{\left(\frac{3n+1}{4n}\right)}^{\frac{n}{n-1}}\left(\frac{u}{\sqrt{kS_{w}\emptyset }}\right)$$

where C is Cannella constant; n is shear coefficient; u is gelant velocity (usually in aqueous phase); k is effective permeability; ∅ is effective porosity; and $S_{w}$ is aqueous phase saturation.

### 2.3. Derivation of Pore Radius

To calculate the average pore radius, we used the Kozeny–Carman model [43], which considers the effective permeability and porosity at a current time step, as shown in Equation (13):

(13) $$r=\frac{1}{2}\sqrt{\frac{\frac{k}{a}{\left(1-\emptyset \right)}^{2}}{\emptyset^{3}}}\;$$

where k is effective permeability; ∅ is effective porosity; and a is proportionality and unit conversion factor.

The conventional model usually ignores the effect of gelant retention on a pore radius. However, this effect cannot be neglected for gelant placement because the gelant retention amount is commonly very high; thus, the size of the pore throat containing the retained gelant could quite likely be reduced. To quantify the effect of retention, we considered a reduced porosity term $\emptyset_{r}$ in Equation (14).

(14) $$\emptyset =\emptyset_{0}-\emptyset_{r}$$

where $\emptyset_{0}$ is original porosity.

The reduced porosity is calculated using Equation (15).

(15) $$\emptyset_{r}=\emptyset_{0}\times \left(C_{ads}\times \frac{M_{w}}{\rho_{p}}\right)$$

where $C_{ads}$ is adsorbed concentration of gelant that is calculated following the Langmuir isothermal in Equation (16).

(16) $$C_{ads}=\left(tad1+tad2*C_{sal}\right)\times \frac{C_{p}}{1+tad3*C_{p}}$$

where tad1,tad2, and tad3 are tuning factors, and $C_{sal}$ is effective salinity.

The retention of gelant can also reduce the effective permeability of the formation. To quantify this reduced permeability, Equation (17) considers a residual resistance factor ($F_{rr}$) on permeability.

(17) $$k=k_{0}*{\left(1+\left(F_{rr}-1\right)\times \frac{C_{ad}}{C_{ad,m}}\right)}^{-1}$$

where $k_{0}$ is original permeability as a function of original porosity and $F_{rr}$ is measured in lab at the retention equilibrium state that has a maximum adsorbed gelant concentration $C_{ad,m}$.

## 3. Overall Diagram of IAPV Model

**Figure 2:**
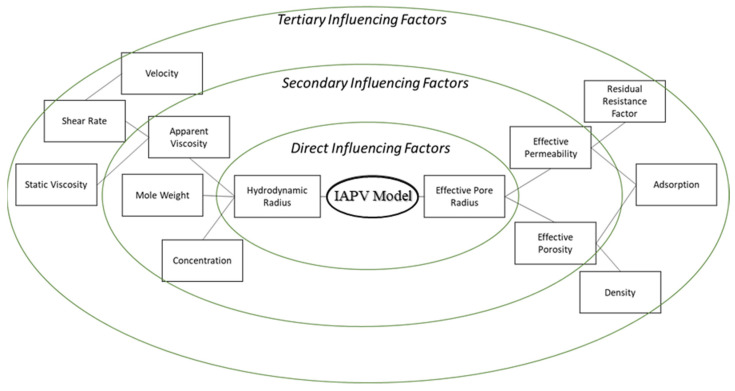
Illustration of factors that influence the gelant IAPV model.

To summarize our novel IAPV model, Figure 2 shows a diagram that displays influential factors on the IAPV. The inner green circle contains these factors, which include the hydrodynamic radius and effective pore radius, both of which directly influence the IAPV based on the theoretical thermal dynamic model. The middle green circle rounds the secondary factors, including apparent viscosity, mole weight, concentration, effective permeability, and porosity, which influence gelant hydrodynamic radius and pore radius. The outer green circle concludes that the extended factors influence the secondary factors. These factors include static viscosity, shear rate or velocity, residual resistance factor, adsorption, mole weight, and density. Deployment of the new model requires the calculations of factors from outer circles to inner circles.

## 4. Model Validation

To validate our new model, we collected IAPV data from 64 experiments [17,24,25,26,44,45,46,47,48,49,50,51,52,53,54]. The influential factors we collected included static viscosity, shear rate (or converted velocity), mole weight, concentration, effective permeability/porosity, adsorption (or retention), residual resistance factor, and density.

Table 1 lists the descriptive data and presents the minimum value (min), maximum value (max), standard deviation (std), mathematical average value (mean), number of samples (count), and box plot. The box plot shows the distribution of the feature samples. The three values shown on the right-hand side of the “box” denote the values of the lower quartile (25% of sample values were below this value), median (50% of sample values were below this value), and upper quartile (75% of sample values were below this value).

For IAPV, it shows that the values ranged from 0 to 49 with a std 9.98, indicating a wide distribution. Absolute permeability Ka ranged from 30 md to 7683 md. Some samples’ values were likely outliers in concentration (conc.), intrinsic viscosity ($\mu_{0}$), residual resistance factor (Frr), retention, and flow velocity, which fell apart from the box range. However, these values were still in the range of this study for gelant. Therefore, we did not exclude these samples from our analysis.

**Figure 3:**
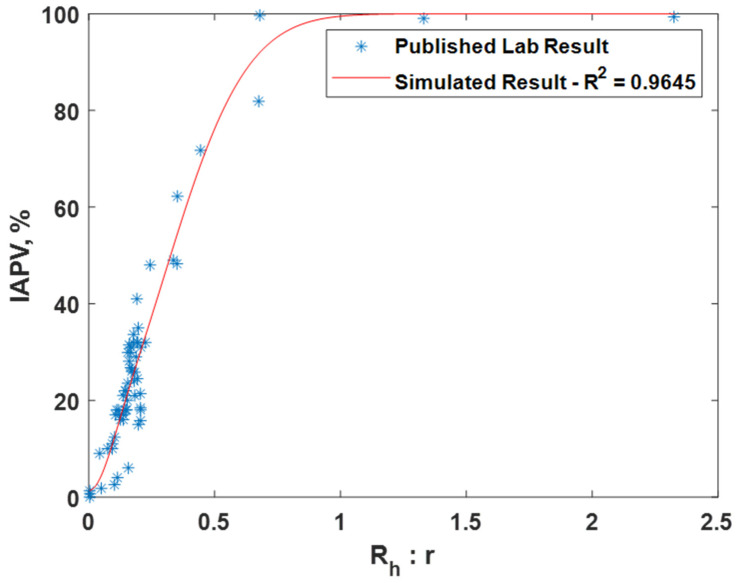
Comparison of calculated IAPV in percentage and published lab results.

The simulated results (Marked as red curve) using our model showed a great match with the published IAPV values measured in prior lab experiments (Marked as blue asterisks), as shown in Figure 3. The values of tuning parameters and constants are shown in Table 2.

## 5. Sensitivity Analysis of Dynamic Factors on IAPV

The major problem of the previous constant model is that it cannot quantify the dynamic change of IAPV during gelant placement. In this section, we tested the sensitivity of dynamic features on IAPV based on the new model. The considered dynamic features include flow velocity, retention, and the combined effect.

### 5.1. Effect of Flow Velocity

During gelant placement, the velocity distribution in reservoirs is commonly not uniform. Due to radial flow regime, flow velocity is relatively higher near wellbore than that in the far field, especially for a vertical well system. Gel treatment is commonly applied in severe heterogenous reservoir that contains fractures. At a high flow velocity, the gelant can have different rheology responses in the matrix and in fractures [14].

**Figure 4:**
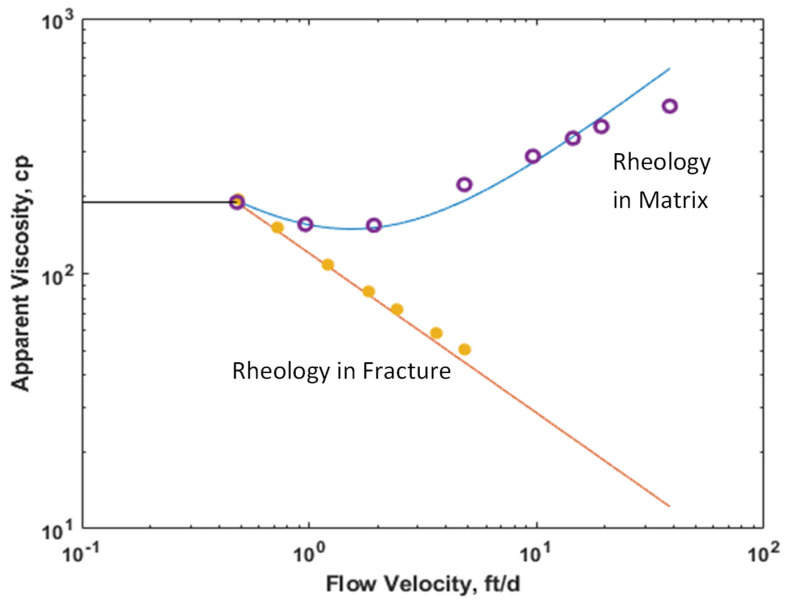
Rheology difference during gelant placement in the matrix and fracture.

To test the sensitivity of flow velocity on IAPV, gelant rheology was considered. We provided a set of gelant rheology data, as shown in Figure 4. Due to the unstable viscosity of gelant mixtures, the rheology data (orange solid circle: in fracture; and purple circle: in matrix) was measured using 5000 ppm HPAM polymer, which was very similar to the gelant at injection stage. The rheology response in the matrix was measured in the coreflooding experiment, and the rheology response in fracture was measured using a viscometer. The static viscosity (black curve) equaled to 190.36 cp, which was measured at the lowest flow velocity, 0.48 ft/d. Curves are simulated results fitting the lab results.

**Figure 5:**
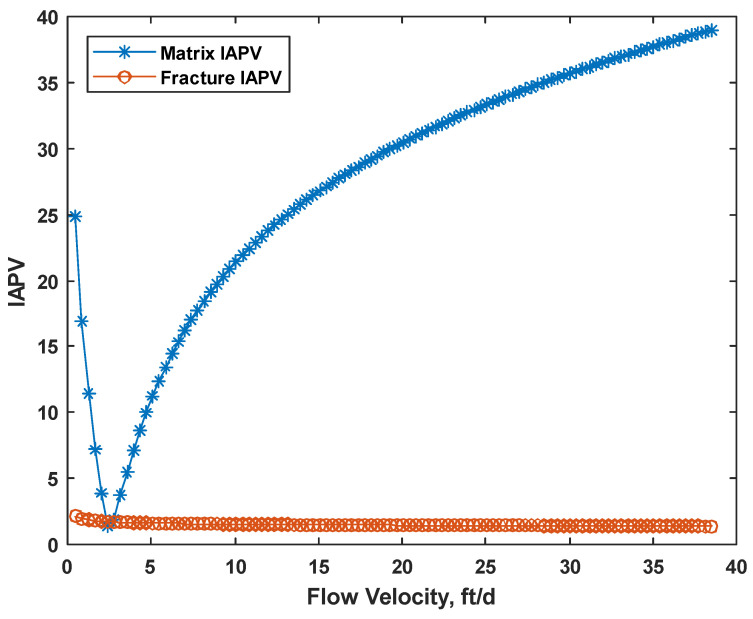
Effect of flow velocity on IAPV in percentage.

We tested the range of velocity from 0.48 ft/d to 38.49 ft/d. Considering constant adsorption 200 μg/g with Frr = 1.5 (no gel formed), K ratio = 100, MW = 20 MMD, the IAPV results of both the matrix and fracture are shown in Figure 5. We observed that the matrix IAPV increased quickly at a high flow velocity period, but the fracture IAPV did not significantly increase in the fracture.

As shown in Figure 5, the relationship between IAPV and flow velocity was nonlinear, which resulted from the power law model of rheology. For IAPV in the matrix, it was very sensitive to the flow velocity. This is because of the varied rheology response in porous media; gelant behaves likes shear thinning at a low shear rate but like shear thickening at a medium to high shear rate. During the shear thinning period, the hydrodynamic radius of gelant decreased when the flow velocity increased, which benefits gelant flowing through the pore throats. Meanwhile, during the shear thickening period, the gelant molecules were elongated by the complex pore networks and viscoelastic nature of the HPAM polymer, which caused elastic turbulence and increased the hydrodynamic radius of gelant. This negatively influenced the gelant flowing through the pore throats. Thus, the IAPV decreased at a low velocity and quickly increased at medium to high velocities.

### 5.2. Effect of Retention

**Figure 6:**
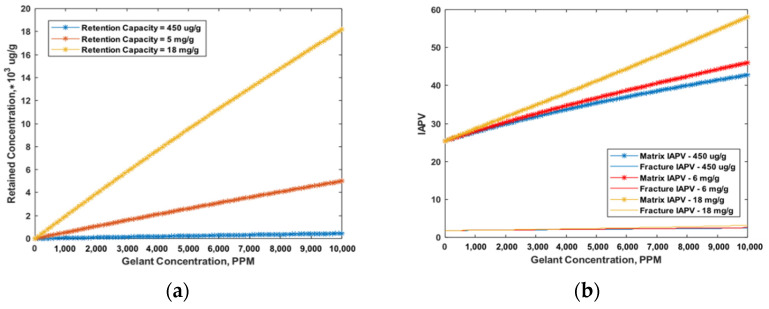
Effect of gelant retention capacity on IAPV. (**a**) Input cases setup; (**b**) Results of IAPV in percentage.

**Figure 7:**
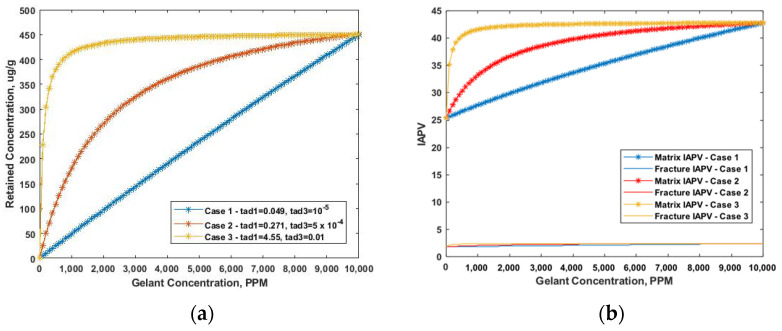
Effect of gelant retention rate on IAPV. (**a**) Input cases setup with varied Langmuir model coefficients; (**b**) Results of IAPV in percentage.

Due to gelant retention, the pore radius was reduced during placement. Thus, the IAPV dynamically increased. Based on Equation (16), we observed that the retention was influenced by both the maximum retained concentration and retention rate. Therefore, we made two sets of test cases and assumed: Frr = 1.5 with no gel formed, constant flow velocity, K ratio = 100, and MW = 20 MMD. We tested the range of the maximum retained concentration from 50 μg/g to 450 μg/g, especially considering constant retention, as shown in Figure 6a. The IAPV results are shown in Figure 6b. Secondly, we tested the effect of the retention rate by considering different Langmuir model’s coefficients. The test cases are shown in Figure 7a and the IAPV results are shown in Figure 7b.

During gelant placement, the effective porosity decreased due to gelant retention. Figure 6 shows the effect of retention capacity on IAPV. We observed that even if a linear retention (retained concentration increased linearly with gelant concentration) was applied, the IAPV nonlinearly increased. Moreover, Figure 6b shows that for the relative low level of retention capacity (blue and red line), the increasing rate of IAPV for the high gelant concentration mildly decreased, yet for the high level of retention capacity (yellow line), the increasing rate increased for the high gelant concentration. This was due to the numerical feature of Equation (13), which indicates that, for higher retention capacity gel, the IAPV can exponentially increase.

Figure 7 shows the effect of different retention rates on IAPV in the Langmuir model. The results show that the increasing rate of IAPV was similar to that of retention concentration when gelant concentration increased. This result indicates that the variation of IAPV was strongly related to gelant retention with a variation of gelant concentration.

### 5.3. Combined Effect on IAPV

**Figure 8:**
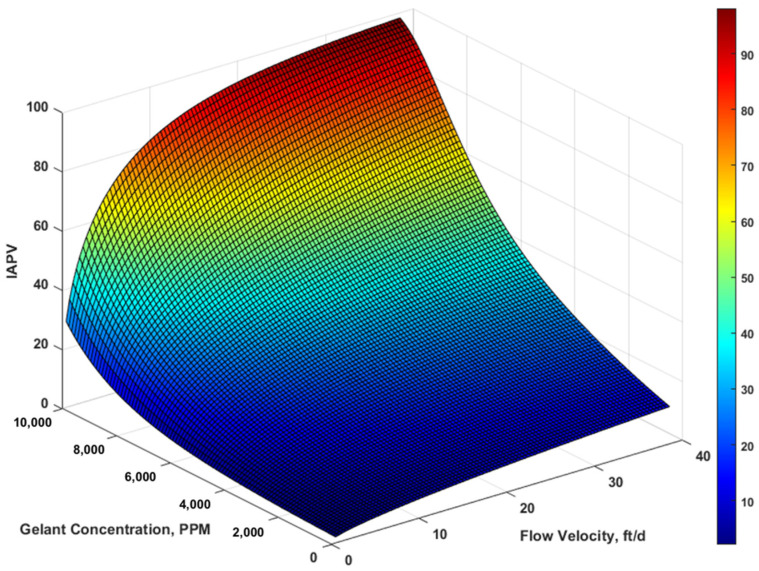
Combined effect on the matrix IAPV results in percentage.

We tested the effect of both retention and flow velocity. We considered concentration variation from 0 to $10^{4}$ PPM with case 3’s retention rate and capacity, as well as the velocity variation from 0.48 to 38 ft/d. The IAPV results are shown in Figure 8 andFigure 9.

To investigate the combined effect of rheology and retention, we made a 3D surface plot using MATLAB. The horizontal axis included a flow velocity range of 0.48 to 38 ft/d and a gelant concentration range from 0 to 10,000 PPM. We observed (Figure 8 andFigure 9) that the matrix IAPV exponentially increased when gelant concentration increased, but logarithmically increased when flow velocity increased. The fracture IAPV did not significantly increase, which only demonstrates the rise of maximum concentration and flow velocity. The results were consistent with our previous analysis.

## 6. Effect of Dynamic IAPV on Gelant Placement

We also investigated the effect of IAPV on gelant placement by considering dynamic features. A conceptual linear flow model was assumed with a low permeability layer (e.g., matrix) and a high permeability layer (e.g., fracture), as shown in Figure 10.

The permeability contrast (high:low) was 100:1. No crossflow was considered. In order to evaluate gelant penetration, we applied a theoretical calculation of the penetration degree (penetration in the low *k* layer $L_{low}$ divided by penetration in the high *k* layer $L_{high}$), as shown in Equation (18). The detailed derivation of this equation was previously mentioned by Seright [9]. In this study, we considered the situation that gelant in the high *k* layer reached the outlet (e.g., $L_{high}$ = 1), so that the degree of penetration offered the penetration of gelant into the low *k* layer.

(18) $$\frac{L_{low}}{L_{high}}=\frac{{\left[1+\left(\mu_{l}-1)\times (\mu_{h}+1\right)\left(\frac{\emptyset_{h}k_{l}}{\emptyset_{l}k_{h}}\right)\left(\frac{1+C_{ads_{h}}-{\mathrm{IAPV}}_{h}}{1+C_{ads_{l}}-{\mathrm{IAPV}}_{l}}\right)\right]}^{0.5}-1}{\mu_{l}-1}$$

where subscription l refers to the low permeability layer and *h* refers to the high permeability layer. We assumed that retention concentrations were the same in the matrix and fracture.

We tested six cases of varied velocity, retention on IAPV, and their combined effects on gelant placement. The cases are listed in Table 3. We applied the Case 1 retention profile for simplification.

The placement results are shown in Figure 11a–i. Figure 11a–c presents the gelant placement results of case 1 to 3. As velocity increased for low concentration gelant, IAPV in the low k layer increased from 27.07 to 96.92, while IAPV in the high k layer increased from 1.84 to 8.80. The difference increased from 25.22 to 88.12. Correspondingly, the degree of penetration decreased from 0.0857 to 0.0047, which was approximately 18 times that of the reduction.

Similarly, we compared cases 4 and 6, as well as cases 7 and 9. We concluded that the degree of penetration was approximately reduced by 12 and 9 times, respectively. These results were consistent with previous analyses that confirmed that when velocity increased, IAPV also increased and induced a significant reduction of the un-preferred penetration into the low k layer.

Comparing the results to another dimension, such as in cases 1, 4, and 7, it is clear that IAPV increased and penetration degree decreased as gelant concentration increased. However, the increasing magnitude was limited. The IAPV increased from 25.22 to 38.53 and the degree of penetration decreased from 0.0857 to 0.0835. For comparison, among cases 3, 6, and 9, the degree of penetration actually increased by 0.0045. The reason for this increased penetration was a result of the retention increase in the high k layer, which increased the IAPV in the high k layer. Concurrently, the IAPV in the low k layer was close to 100%, thus it did not have much space to increase. Consequently, the difference between the IAPV in the high and low k layers was reduced from 88.12 to 85.04, which limited its benefit to gelant placement.

## 7. Discussions

As discussed above, to effectively quantify the IAPV, we considered not only the influential factors on IAPV but also the consequent effects on gelant placement. However, previous studies tended to consider the consequences of IAPV while ignoring the influential factors on IAPV, especially the dynamic factors. Moreover, the concept of IAPV contains microscopic and macroscopic phenomena [55]. However, for gel simulation, the field scale reservoir simulation approaches often overlooked the microscale solid–fluid interactions, while experimental inquiries are plagued by high costs and limited resolutions [56]. Thus, a comprehensive characterization of IAPV in reservoir simulations was a challenge.

The novel IAPV model developed in this paper establishes an innovative approach to bridge microscopic and macroscopic features. For microscopic features, this model can evaluate pore occupation, pore radius variation, and gelant elongation, while for macroscopic features, this model can quantify the permeability variation and gelant rheology. As a result, the model can be integrated into reservoir simulators based on Darcy’s Law and continuity equations.

In recent decades, many field applications reported that large amounts of in situ gel could be successfully placed in fractures without significantly damaging the matrix [57]. As a result, a number of simulation studies have tried to simulate the preferential penetration mechanisms during gelant placement. However, IAPV has never been considered to be an important factor because of the ineligible model [35].

**Figure 9:**
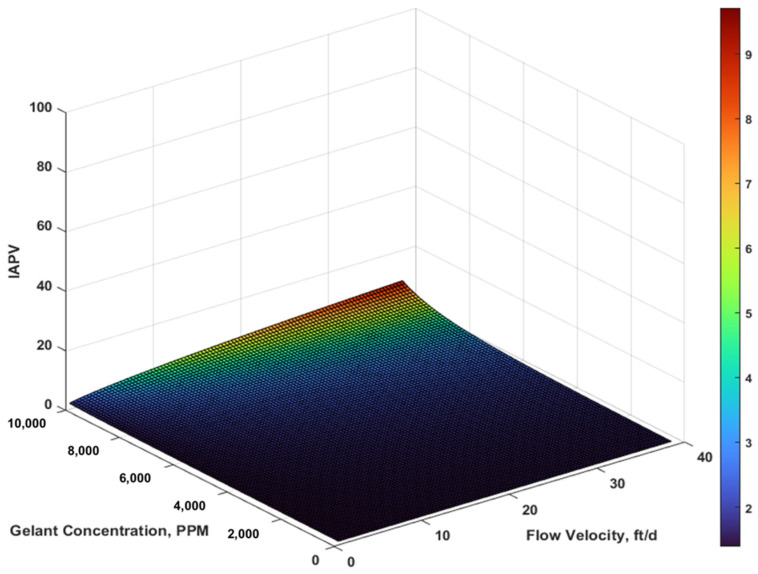
Combined effect on fracture IAPV in fracture in percentage.

**Figure 10:**
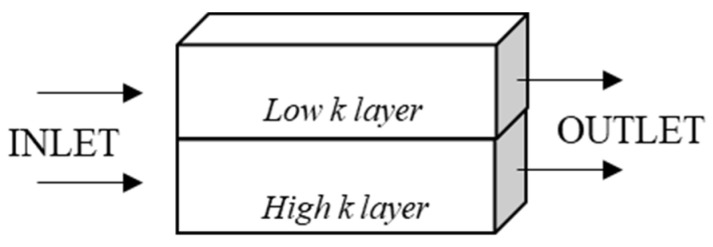
Conceptual linear flow model illustration.

In fact, the impact of IAPV is often underestimated because of the ignorance of dynamic characterization. As shown in Figure 8 and Figure 9, the IAPV varied greatly with variation of gelant concentration and flow velocity. Because of heterogeneity, the fluid flow in the matrix commonly experiences lower flow velocity than that in fractures. Moreover, because of the different flow regime, the flow velocity near the wellbore radial flow region can experience a higher velocity than the far field linear flow region. Due to the radial flow, the near wellbore had much higher gelant concentrations than that of the far field. Consequently, by combining these features, we concluded that, for the near wellbore matrix, where flow velocity and gelant concentration were higher than for the far field, the IAPV was most likely to reach the maximum value.

**Figure 11:**
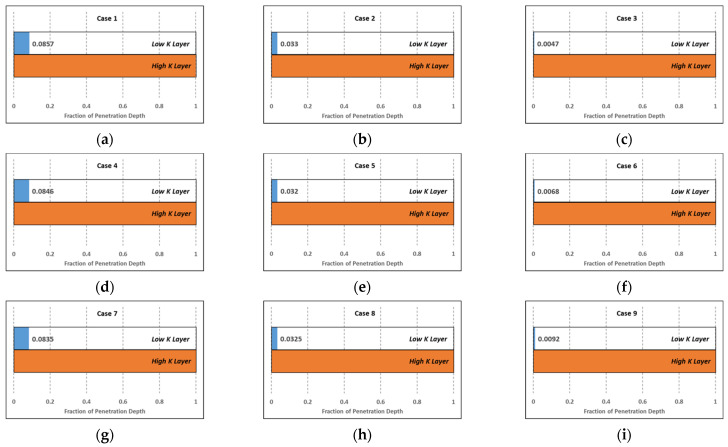
Gelant placement results in the low k and high k layers in the new IAPV model (sub-figure (**a**–**i**) corresponding to Cases 1–9).

Practically, the large value of IAPV greatly decreased the effective porosity of gelant in the matrix and thus reduced the un-preferred penetration of gelant into the matrix. The calculated gelant placement results using our new IAPV model are shown in Figure 11. These results show how the IAPV influences the placements of gelant. Our model demonstrates how the IAPV can effectively decrease gelant penetration in the low k matrix. In the conventional simulation model, the matrix is considered to be fully accessible or partially accessible (with a constant IAPV) to gelant. Thus, the penetration of gelant deep into the matrix can hardly be limited. Due to the high retention and permeability reduction ability of formed gel, the gelant penetration into the matrix is a permanent damage to the oil-bearing zone, which is not preferred.

In fact, extensive lab experiments [58,59,60,61] have shown that after gel placement, a filter cake forms on the matrix surfaces and the water in the gel system leaks into the matrix. This is because of sharp decreasing effective pore volume in the matrix for the gelant or gel when it reaches the matrix surface due to the quickly increasing IAPV. This does not occur for the water in the solution. Consequently, our model is qualified to simulate gelant placement in order to more accurately assess lab results.

## 8. Conclusions

Based on the theoretical thermal dynamic model, a numerical IAPV model was derived and validated by previous lab results as found in the literature. Compared with the conventional constant model in simulators, the new model can quantify indirect and direct effects of both static and dynamic features, which includes a combination of eight factors. Here, a sensitivity test was conducted. The results showed that IAPV in matrix was strongly sensitive to the apparent viscosity that related to flow velocity and the retention that related to gelant concentration. As a result, the consideration of dynamic feature in IAPV model was indispensable. The new model also effectively quantified the impact of IAPV on limiting gelant penetration into the low k layer, especially considering the gelant rheology and retention. Consequently, the higher the flow velocity and gelant concentration, the greater the IAPV benefits were on the degree of penetration. Lastly, the new IAPV model proposed in this paper can play a significant role in simulation of in situ gel treatment in order to understand and simulate the preferential penetration mechanism.

## Figures and Tables

**Table 1:** The descriptive information of the collected data.

Index	IAPV, %	Ka, md	Porosity	Mole Weight, MMdalton	Conc., ppm	Resistance Factor	Frr	Retention, ug/g	Flow Velocity, ft/d
**Count**	64	64	64	64	64	64	64	64	64
**Mean**	22.00	819.76	0.23	10.70	875.78	10.38	3.64	43.72	2.43
**Std**	23.69	1315.34	0.05	7.79	880.57	7.32	2.43	29.56	2.56
**Min**	0.00	30.00	0.12	2.00	49.00	1.00	1.00	9.00	0.10
**Max**	99.36	7683.00	0.41	30.00	5500.00	39.69	16.00	215.00	10.00
**Box Plot**	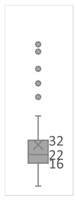	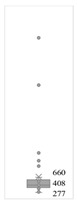	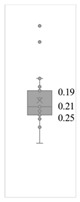	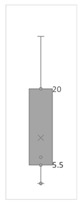	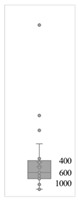	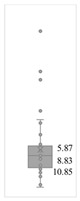	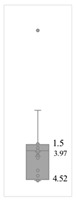	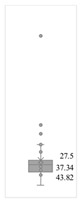	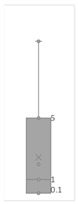

**Table 2:** Tuning parameter and constant value for fitting lab result.

m	3	$\beta_{1}$	−8.005
$\beta_{2}$	2.155	$\beta_{3}$	8.003
$\delta_{1}$	1.052	$\delta_{2}$	0
C	6	tad1	$5\times 10^{-2}$
tad2	0	tad3	$10^{-5}$

**Table 3:** Gelant placement cases setup.

	Velocity, ft/d	Gelant Conc., PPM	IAPV*_l_*	IAPV*_h_*
Case 1	0.48	2000	27.07	1.84
Case 2	5	2000	63.60	3.52
Case 3	38	2000	96.92	8.80
Case 4	0.48	6000	32.77	2.03
Case 5	5	6000	74.27	4.33
Case 6	38	6000	99.19	11.24
Case 7	0.48	10,000	40.87	2.34
Case 8	5	10,000	85.76	5.66
Case 9	38	10,000	99.92	14.87

## References

[1] Sagbana P.I., Abushaikha A.S. (2021). A comprehensive review of the chemical-based conformance control methods in oil reservoirs. J. Pet. Explor. Prod. Technol..

[2] Bai B., Li Y., Liu X. (1999). New development of water shutoff and profile control in oilfields in China. Oil Drill. Prod. Technol..

[3] Bai B., Zhou J., Yin M. (2015). A comprehensive review of polyacrylamide polymer gels for conformance control. Pet. Explor. Dev..

[4] Seright R.S., Lane R.H., Sydansk R.D. A strategy for attacking excess water production. Proceedings of the SPE Permian Basin Oil and Gas Recovery Conference.

[5] Zhao S., Zhu D., Bai B. (2021). Experimental study of degradable preformed particle gel (DPPG) as temporary plugging agent for carbonate reservoir matrix acidizing to improve oil recovery. J. Pet. Sci. Eng..

[6] Zhu D.-Y., Luo R.-T., Liu Y., Qin J.-H., Zhao Q., Zhang H.-J., Wang W.-S., Wang Z.-Y., Zhu M.-E., Wang Y.-P. (2022). Development of re-crosslinkable dispersed particle gels for conformance improvement for extremely high-temperature reservoirs. Pet. Sci..

[7] Sydansk R.D. (1988). A new conformance-improvement-treatment chromium (III) gel technology. Proceedings of the SPE Enhanced Oil Recovery Symposium.

[8] Sydansk R.D. (1990). A newly developed chromium (III) gel technology. SPE Res. Eng..

[9] Seright R.S. (1988). Placement of gels to modify injection profiles. Proceedings of the SPE/OOE Enhanced Oil Recovery Symposium.

[10] Seright R.S., Zhang G., Akanni O.O., Wang D. (2011). A comparison of polymer flooding with in-depth profile modification. Proceedings of the Canadian Unconventional Resources Conference.

[11] Sydansk R.D., Southwell G.P. (2000). More than 12 years of experience with a successful conformance-control polymer gel technology. Proceedings of the SPE/AAPG Western Regional Meeting.

[12] Sydansk R.D., Xiong Y., Al-Dhafeeri A.M., Schrader R.J., Seright R.S. (2005). Characterization of partially formed polymer gels for application to fractured production wells for water-shutoff purposes. SPE Prod. Facil..

[13] Brattekås B., Seright R. (2020). The mechanism for improved polymer gel blocking during low-salinity waterfloods, investigated using positron emission tomography imaging. Transp. Porous Media.

[14] Leng J., Wei M., Bai B. (2022). Impact of Polymer Rheology on Gel Treatment Performance of Horizontal Wells with Severe Channeling. SPE J..

[15] Leng J., Wei M., Bai B., Seright R.S., Zhang Y., Cercone D., Ning S. Impact of Rheology Models on Horizontal Well Polymer Flooding in a Heavy Oil Reservoir on Alaska North Slope: A Simulation Study. Proceedings of the Offshore Technology Conference.

[16] Seright R., Brattekas B. (2021). Water shutoff and conformance improvement: An introduction. Pet. Sci..

[17] Dawson R., Lantz R.B. (1972). Inaccessible Pore Volume in Polymer Flooding. Soc. Pet. Eng. J..

[18] DiMarzio E.A., Guttman C.M. (1970). Separation by Flow. Macromolecules.

[19] Szabo M.T. Micellar Shear Degradation, Formation Plugging, And Inaccessible Pore Volume. Proceedings of the SPE Annual Fall Technical Conference and Exhibition.

[20] Zhang G., Seright R. (2014). Effect of concentration on HPAM retention in porous media. SPE J..

[21] Shah B.N., Lawrence G., Willhite P., Green D.W. The effect of inaccessible pore volume on the flow of polymer and solvent through porous media. Proceedings of the SPE Annual Fall Technical Conference and Exhibition.

[22] Gupta S.P., Trushenski S.P. (1976). Micellar flooding: The design of the polymer mobility buffer bank (No. CONF-761008-87). Proceedings of the Annual Meeting of the Society of Petroleum Engineers.

[23] Ilyasov I., Koltsov I., Golub P., Tretyakov N., Cheban A., Thomas A. (2021). Polymer Retention Determination in Porous Media for Polymer Flooding in Unconsolidated Reservoir. Polymers.

[24] Liauh W.C., Duda J.L., Klaus E.E. (1979). An Investigation of The Inaccessible Pore Volume Phenomena. Proceedings of the 84th National American Institute of Chemical Engineers Meeting.

[25] Pancharoen M., Thiele M.R., Kovscek A.R. (2010). Inaccessible Pore Volume of Associative Polymer Floods. Proceedings of the SPE Improved Oil Recovery Symposium.

[26] Lotsch T., Muller T., Pusch G. (1985). The Effect of Inaccessible Pore Volume on Polymer Coreflood Experiments. Proceedings of the SPE Oilfield and Geothermal Chemistry Symposium.

[27] Gilman J.R., MacMillan D.J. (1987). Improved interpretation of the inaccessible pore-volume phenomenon. SPE Form. Eval..

[28] Sorbie K.S. (1989). Network Modeling of Xanthan Rheology in Porous Media in the Presence of Depleted Layer Effects. Proceedings of the SPE Annual Technical Conference and Exhibition.

[29] Omari A., Moan M., Chauveteau G. (1989). Wall effects in the flow of flexible polymer solutions through small pores G. Rheol. Acta.

[30] Chauveteau G., Zaitoun A. (1981). Basic Rheological Behavior of Xanthan Polysaccharide Solutions in Porous Media: Effects of Pore Size and Polymer Concentration. Proceedings to the European Symposium on EOR.

[31] Ferreira V.H.S., Moreno R.B.Z.L. (2019). Rheology-based method for calculating polymer inaccessible pore volume in core flooding experiments. E3S Web Conf..

[32] Manichand R.N., Seright R.S. (2014). Field vs. laboratory polymer-retention values for a polymer flood in the Tambaredjo field. SPE Reserv. Eval. Eng..

[33] Swadesi B., Zumar R., Sanmurjana M., Siregar S., Kristanto D. (2021). The effect of inaccessible pore volume and adsorption on polymer flooding for field scale injection in RZ field. AIP Conf. Proc..

[34] Fedorov K.M., Pospelova T.A., Kobyashev A.V., Gilmanov A.Y., Kovalchuk T.N., Shevelev A.P. Determination of Adsorption-Retention Constants and Inaccessible Pore Volume for High-Molecular Polymers. Proceedings of the SPE Russian Petroleum Technology Conference.

[35] Bai B., Leng J., Wei M. (2021). A comprehensive review of in-situ polymer gel simulation for conformance control. Pet. Sci..

[36] DiMarzio E.A. (1965). Proper Accounting of Conformations of a Polymer near a Surface. J. Phys. Chem..

[37] Lohne A., Nødland O., Stavland A., Hiorth A. (2017). A model for non-Newtonian flow in porous media at different flow regimes. Comput. Geosci..

[38] Hiemenz P.C., Lodge T.P. (2007). Polymer Chemistry.

[39] Hirasaki G., Pope G. (1974). Analysis of factors influencing mobility and adsorption in the flow of polymer solution through porous media. Soc. Pet. Eng. J..

[40] Delshad M., Kim D.H., Magbagbeola O.A., Huh C., Pope G.A., Tarahhom F. (2008). Mechanistic interpretation and utilization of viscoelastic behavior of polymer solutions for improved polymer-flood efficiency. SPE Symposium on Improved Oil Recovery.

[41] Zechner M., Buchgraber M., Clemens T., Gumpenberger T., Castanier L.M., Kovscek A.R. Flow of Polyacrylamide Polymers in the Near-Wellbore-Region, Rheological Behavior within Induced Fractures and Near-Wellbore-Area. Proceedings of the SPE Annual Technical Conference and Exhibition.

[42] Cannella W.J., Huh C., Seright R.S. (1988). Prediction of xanthan rheology in porous media. Proceedings of the SPE Annual Technical Conference and Exhibition.

[43] Kozeny J. (1927). Ueber kapillare Leitung des Wassers im Boden. R. Acad. Sci. Vienna Proc. Class I.

[44] AlSofi A.M., Wang J., Leng Z., Abbad M., Kaidar Z.F. Assessment of polymer interactions with carbonate rocks and implications for EOR applications. Proceedings of the SPE Kingdom of Saudi Arabia Annual Technical Symposium and Exhibition.

[45] Dominguez J.G., Willhite G.P. (1977). Retention and Flow Characteristics of Polymer Solutions in Porous Media. Soc. Pet. Eng. J..

[46] Fletcher A.J.P., Flew S.R.G., Lamb S.P., Lund T., Bjornestad E., Stavland A., Gjovikli N.B. (1991). Measurements of Polysaccharide Polymer Properties in Porous Media. Proceedings of the SPE International Symposium on Oilfield Chemistry.

[47] Gupta S.P. (1978). Micellar Flooding the Propagation of the Polymer Mobility Buffer Bank. Soc. Pet. Eng. J..

[48] Hatzignatiou D.G., Moradi H., Stavland A. (2013). Experimental Investigation of Polymer Flow through Water- and Oil-Wet Berea Sandstone Core Samples. Proceedings of the EAGE Annual Conference & Exhibition Incorporating SPE Europec.

[49] Huang Y., Sorbie K.S. (1993). Scleroglucan Behavior in Flow Through Porous Media: Comparison of Adsorption and In-Situ Rheology Withwith Xanthan. Proceedings of the SPE International Symposium on Oilfield Chemistry.

[50] Hughes D.S., Teeuw D., Cottrell C.W., Tollas J.M. (1990). Appraisal of the Use of Polymer Injection to Suppress Aquifer Influx and To Improve Volumetric Sweep in a Viscous Oil Reservoir. Soc. Pet. Eng. Reserv. Eng..

[51] Knight B.L., Rhudy J.S. (1977). Recovery Of High-Viscosity Crudes by Polymer Flooding. J. Can. Pet. Technol..

[52] Osterloh W.T., Law E.J. (1998). Polymer Transport and Rheological Properties for Polymer Flooding in the North Sea. Proceedings of the SPE/DOE Improved Oil Recovery Symposium.

[53] Trushenski S.P., Dauben D.L., Parrish D.R. (1974). Micellar Flooding—Fluid Propagation, Interaction, and Mobility. SPE J..

[54] Vela S., Peaceman D.W., Sandvik E.I. (1976). Evaluation of Polymer Flooding in a Layered Reservoir with Crossflow, Retention, and Degradation. SPE J..

[55] Braconnier B., Nguepi G., Preux C., Tran Q., Berthon C. (2022). Towards Weakly Hyperbolic Models for Inaccessible Pore Volume in Polymer Flooding. https://hal.archives-ouvertes.fr/hal-03627845.

[56] Cook B.K. (2001). A Numerical Framework for the Direct Simulation of Solid-Fluid Systems. Doctoral Dissertation.

[57] Seright R., Zhang G., Akanni O., Wang D. (2012). A comparison of polymer flooding with in-depth profile modification. J. Can. Pet. Technol..

[58] Brattekås B., Seright R., Ersland G. (2020). Water leakoff during gel placement in fractures: Extension to oil-saturated porous media. SPE Prod. Oper..

[59] Wang Z., Bai B., Sun X., Wang J. (2019). Effect of multiple factors on preformed particle gel placement, dehydration, and plugging performance in partially open fractures. Fuel.

[60] Zhao Y., Bai B. (2022). Experimental study of transport behavior of swellable microgel particles in superpermeable channels for conformance control. SPE J..

[61] Zhao Y., Bai B. (2022). Selective penetration behavior of microgels in superpermeable channels and reservoir matrices. J. Pet. Sci. Eng..

